# The *Citrobacter freundii* Complex as an Emerging Pathogen: Genomic Plasticity, Virulence, and Antimicrobial Resistance

**DOI:** 10.3390/ijms27052378

**Published:** 2026-03-04

**Authors:** Anca-Elena Duduveche

**Affiliations:** Department of Infectious Diseases, University of Medicine and Pharmacy of Craiova, 200349 Craiova, Romania; anca.duduveche@umfcv.ro; Tel.:+40-721219954

**Keywords:** *Citrobacter freundii*, pathogen, virulence, genomic plasticity, antimicrobial resistance, infections

## Abstract

The *Citrobacter freundii* (*C. freundii*) complex represents an increasingly significant group of opportunistic pathogens within healthcare settings. This bacterial complex demonstrates remarkable genomic plasticity, characterized by extensive horizontal gene transfer capabilities that facilitate rapid acquisition of resistance determinants and virulence factors. Although originally considered environmental organisms with limited pathogenic potential, members of the *C. freundii* complex have emerged as important nosocomial pathogens responsible for urinary tract infections, bacteremia, wound infections, and neonatal meningitis. Importantly, their clinical significance lies less in unique disease manifestations and more in the moderate risk of resistance emergence during therapy with third-generation cephalosporins, driven by inducible chromosomal AmpC β-lactamase production. Beyond this intrinsic mechanism, the genomic adaptability of the *C. freundii* complex also enables acquisition of additional resistance determinants, including extended-spectrum β-lactamases (ESBLs) and carbapenemases, further limiting therapeutic options and complicating clinical management. Understanding the molecular mechanisms underlying genomic plasticity, virulence expression, and resistance development in the *C. freundii* complex is crucial for developing effective diagnostic strategies, infection control measures, and novel therapeutic approaches. This pathogen exemplifies the challenge of emerging multidrug-resistant bacteria in contemporary healthcare and underscores the need for continued surveillance and research. This narrative review provides current insights into the taxonomy, genomic plasticity, virulence, and mechanisms of antibiotic resistance.

## 1. Introduction

The *Citrobacter freundii* complex is increasingly implicated in urinary tract and nosocomial infections, particularly among elderly patients with comorbidities and prolonged exposure to hospital environments or invasive procedures [1,2,3,4]. Unlike *Escherichia coli*, which dominates community-acquired urinary tract infections, *C. freundii* is associated with complicated, polymicrobial infections, often of abdominal or post-surgical origin, and with a significant proportion of nosocomial cases [3,5,6]. The underlying justification for the observed increase in incidence can be attributed to genomic plasticity alongside the capacity to acquire and disseminate resistance genes, including extended-spectrum beta-lactamases (ESBLs) and carbapenemases (e.g., blaNDM-1), which promote the emergence of multidrug-resistant strains that present significant therapeutic challenges [1,4,7,8]. Such strains possess the ability to colonize and persist within hospital reservoirs (e.g., sinks, drains), thereby facilitating indirect transmission to patients, as substantiated by genomic typing investigations and outbreak analyses [2,9]. From a clinical perspective, infections caused by the *C. freundii* complex correlate with heightened morbidity, extended duration of hospital admission, and considerable mortality rates among susceptible patient populations [1,3,5]. The presence of resistance to third-generation cephalosporins and carbapenems severely constrains therapeutic alternatives, necessitating rigorous epidemiological surveillance and stringent infection control protocols [1,2,7]. This review intends to provide a thorough overview of *Citrobacter freundii*, emphasizing its genomic plasticity, virulence factors, and patterns of antimicrobial resistance.

## 2. Taxonomy and Genomic Organization of *C. freundii* Complex

The *Citrobacter freundii* complex has been historically misidentified due to constraints in phenotypic assessment, which amalgamates various *Citrobacter* species exhibiting analogous biochemical characteristics under the designation *C. freundii*, consequently obfuscating their authentic genomic heterogeneity. This misclassification has resulted in ambiguity regarding the distinctions among *C. freundii*, *C. braakii*, *C. portucalensis*, and other phylogenetically affiliated species, with significant ramifications for the epidemiology, therapeutic interventions, and monitoring of infectious diseases [10,11,12].

Conventional phenotypic assays have a species-level correct identification rate of approximately 46.8% for *Citrobacter* spp., with a significant rate of misidentification between species [11,13]. MALDI-TOF MS (Matrix-Assisted Laser Desorption Ionization Time-of-Flight Mass Spectrometry), although much better than phenotypic methods, still has limitations at the species level for the *Citrobacter freundii* complex, especially when reference spectra in the database are incomplete or when atypical strains are present. Recent studies show that the misidentification rate with MALDI-TOF MS is approximately 16.1% (83.9% species-level correct identification for *Citrobacter* spp.), compared to whole-genome sequencing (WGS), which provides 100% accuracy [12,13,14]. WGS, using average nucleotide identity (ANI) analysis and genomic phylogeny, allows for precise identification and re-classification of *Citrobacter* strains, correcting errors in the MALDI-TOF MS and GenBank databases, and highlighting the existence of distinct species within the *C. freundii* complex [9,12,14]. Thus, for clinical or epidemiological cases where species-level identification is critical, WGS is the reference method, and MALDI-TOF MS remains useful for rapid screening, with recognized limitations [13,14].

The current species included in the *Citrobacter freundii* complex are: *Citrobacter freundii*, *Citrobacter braakii*, *Citrobacter youngae*, *Citrobacter pasteurii*, *Citrobacter werkmanii*, and *Citrobacter portucalensis* [15,16,17]. These congeners were delimited based on genomic analysis, as phenotypic methods and 16S rRNA sequencing do not allow for reliable discrimination at the species level [16,17]. The genomic similarity threshold (ANI) used to delimit species in the *Citrobacter freundii* complex is ≥95% ANI between genomes, with a genome alignment fraction of at least 70% [18,19,20]. Values below this threshold indicate membership in different species. In addition, phylogenomic criteria are also used, as well as core-genome analysis and multilocus sequence analysis (MLSA) on conserved genes (e.g., rpoB, fusA, pyrG, leuS), which confirm species separation when corroborated with ANI values [9,15]. For taxonomic validation, it is recommended to combine ANI with digital DNA-DNA hybridization (dDDH), where the speciation threshold is approximately 70% dDDH, and with core-genome phylogenetic analysis [16,18].

**Figure 1:**
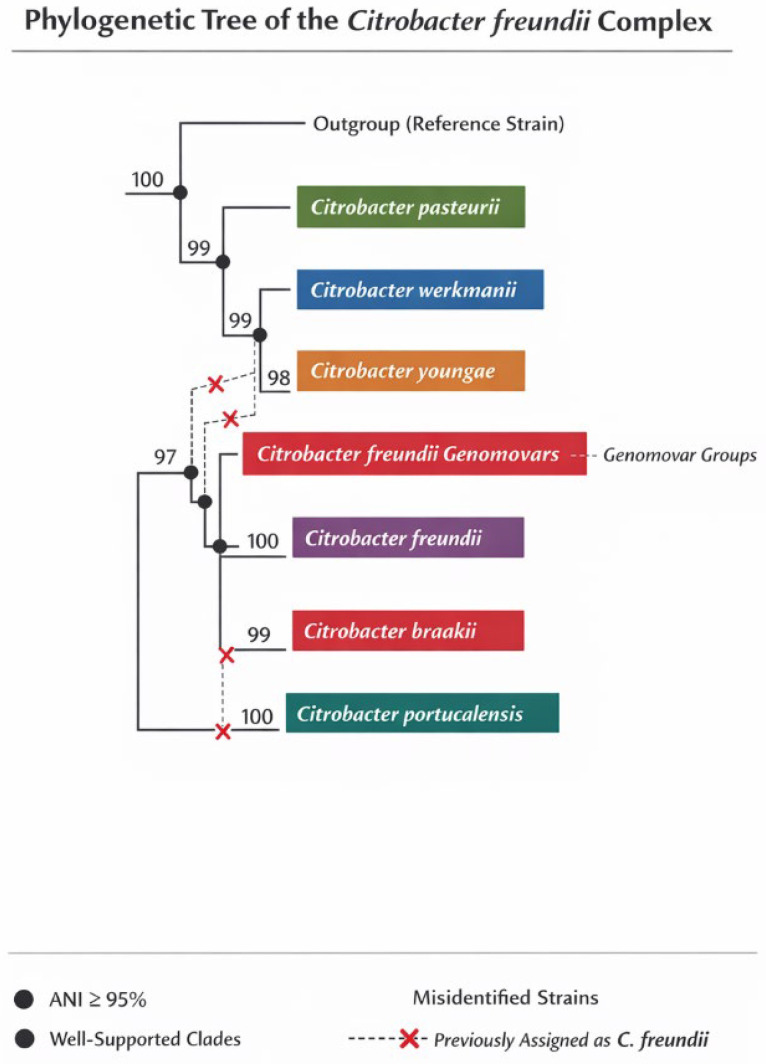
Shows the phylogenetic tree of the species within the *Citrobacter freundii* complex, illustrating the distinct genetic relationships and evolutionary divergence among the currently recognized species in the complex, including *Citrobacter freundii*, *C. braakii*, *C. youngae*, *C. pasteurii*, *C. werkmanii*, and *C. portucalensis*. The tree demonstrates that these species form well-supported, separate clades, confirming their status as independent species rather than subtypes, based on whole-genome sequence analysis and average nucleotide identity (ANI) thresholds of ≥95%. The phylogenetic structure also highlights the presence of multiple genomovars and reveals that some strains previously classified as *C. freundii* are misassigned, supporting the need for taxonomic reclassification. Additionally, the tree shows that species boundaries within the complex cannot be reliably distinguished by traditional phenotypic or single-gene markers, but require comprehensive phylogenomic approaches for accurate identification.

Figure 1 depicts the phylogenetic tree of the *Citrobacter freundii* complex, illustrating genetic relationships and evolutionary divergence among recognized species. The tree confirms their independent status as species based on whole-genome sequence analysis and ANI thresholds of ≥95%. It also indicates the presence of multiple genomovars and suggests misassignment of some strains as *C. freundii*, necessitating taxonomic reclassification. Furthermore, it emphasizes that traditional phenotypic or single-gene markers are insufficient for distinguishing species boundaries, advocating for comprehensive phylogenomic approaches.

The pangenome architecture of the *Citrobacter freundii* complex is distinguished by its open pangenome, which showcases substantial genetic variability and an ongoing potential for the assimilation of novel genes via horizontal gene transfer [21,22]. The core genome comprises conserved genes that play critical roles in fundamental biological processes, including central metabolic pathways (such as diversified nutrient catabolism, denitrification, and ammonification), responses to oxidative and osmotic stress, as well as biofilm regulation, thereby imparting metabolic flexibility and adaptability to diverse ecological niches [21,23]. These genes facilitate the effective utilization of carbon and energy substrates, confer resilience to environmental perturbations, and enhance survival in challenging conditions, particularly within healthcare settings. The accessory genome consists of resistance islands and virulence clusters, located in hypervariable regions separated by the synthesis blocks of the genome [1,2]. These regions contain genes for carbapenemases, ESBLs, efflux pumps, integrons, IncC/IncM2/IncP6 plasmids, as well as virulence genes (secretion systems, toxins, adhesion factors, and biofilm) [21,24]. The accessory genome is responsible for the rapid emergence of multidrug-resistant strains and the phenotypic diversity of virulence.

## 3. Environmental and Reservoir Biology

The *Citrobacter freundii* complex has a versatile ecological profile, being frequently identified in hospital sinks and drains, wastewater, soil, and sediments. In the hospital environment, sinks and drains represent persistent reservoirs, favoring indirect transmission to patients, with a major role in the epidemiology of nosocomial infections. Multiple investigations have documented hospital clusters involving bloodstream infections, neonatal units, and intensive care settings, often linked to contaminated sinks, medical equipment, or environmental reservoirs [24,25,26]. These outbreaks underscore the organism’s ability to survive in moist hospital niches, acquire resistance determinants, and spread clonally among vulnerable patients. The recognition of CFC as a potential outbreak-associated pathogen reinforces the need for active surveillance, rigorous environmental hygiene, and careful antimicrobial stewardship. In wastewater, including that originating from hospitals, *Citrobacter* spp. are constantly detected, often as multidrug-resistant strains [9,27,28,29]. Adaptation to heavy metals and disinfectants is supported by the presence of resistance genes to copper, chromium, silver, as well as to biocides (e.g., benzalkonium, cetylpyridinium), located on mobile elements (plasmids, transposons) [30]. Exposure to high concentrations of metals and biocides in the hospital environment and in sewage treatment plants selects resistant strains that can survive disinfection and treatment procedures [25,30].

Biofilm formation in aquatic systems is a key feature that facilitates persistence and protection against environmental stress and antimicrobials [26,27,31]. The *Citrobacter* biofilm is composed of extracellular polymeric substances (EPSs), including polysaccharides, proteins, extracellular DNA, and lipids, with compositional variations depending on the environment and selective pressure (e.g., the presence of antibiotics or biocides) [27,31]. EPSs confer resistance to disinfectants and favor horizontal transfer of resistance genes [27]. Persistence is supported by adaptive mutations affecting stress response systems, biofilm regulation, efflux pumps, and modification of membrane porins [10,30]. The accessory genome, with resistance islands and virulence clusters, allows rapid acquisition of new genes, facilitating adaptation to hostile environments and antimicrobial treatments [10,24,30].

In addition to environmental niches, the gastrointestinal tract of humans and animals represents an important natural reservoir for the *Citrobacter freundii* complex. Studies of fecal microbiota from healthy individuals have demonstrated frequent colonization by genetically diverse strains harboring multiple chromosomal AmpC variants [32]. The marked polymorphism observed among β-lactamase genes within commensal populations suggests ongoing evolutionary adaptation under antimicrobial and ecological pressures. Notably, *C. freundii* may persist long-term as a gut commensal, creating opportunities for accumulation and horizontal dissemination of resistance determinants to more pathogenic Enterobacterales [32]. This intestinal reservoir further supports the role of the CFC as both a reservoir and vector of antimicrobial resistance within the One Health continuum.

## 4. Virulence and Pathogenesis: What We Know and What Is Missing

The principal virulence determinants of the *Citrobacter freundii* complex that play a crucial role in adhesion and colonization are the FimH-type adhesins and fimbrial systems, which encompass type IV pili. FimH, situated at the terminal end of the type 1 fimbria, facilitates the recognition and binding of mannosylated glycoproteins present on the surface of host cells, thereby promoting the colonization of the urinary tract and the formation of biofilms [33]. The allelic variation in FimH affects both binding affinity and tissue tropism, which in turn influences bacterial virulence and persistence [33].

Type IV pili facilitate adhesion to abiotic surfaces (such as sinks and medical devices) as well as epithelial cells, and they play a significant role in biofilm formation and maturation, motility, and horizontal gene transfer. The structural characteristics and functional capabilities of these pili are modulated by specific genomic loci, accompanied by evolutionary adaptations that enable integration into a variety of bacterial environments and lifestyles [34,35].

Current gaps in understanding these mechanisms include incomplete characterization of the structural and functional diversity of adhesins and pili at the species and strain levels, as well as how the regulation of expression of these factors influences pathogenicity in the clinical context. The ramifications of the interplay among adhesins, pili, and various secretion systems on virulence and therapeutic resistance remain inadequately elucidated [36,37,38]. Additional investigations are imperative to elucidate the precise function of these structures in nosocomial infections and to ascertain prospective therapeutic targets.

The enterobactin and aerobactin systems represent the principal mechanisms for iron acquisition within the *Citrobacter freundii* complex, which are critical for the bacterium’s survival and pathogenicity in the host milieu, wherein free iron is sequestered by host proteins. Enterobactin serves as a catecholate siderophore exhibiting an exceptionally high affinity for Fe(III), facilitating the effective uptake of iron from the extracellular environment and its translocation into the cell via TonB-dependent systems and specialized receptors [39,40,41]. Aerobactin, characterized as a citrate-hydroxamate siderophore, fulfills a complementary function, being linked to enhanced virulence, biofilm formation, and resistance to oxidative stress, particularly under conditions of iron scarcity [42,43]. Iron-regulated virulence transcriptional networks are orchestrated by the ferric uptake regulator (Fur), which inhibits the expression of siderophore biosynthesis and transport genes in the presence of iron and activates them under iron deficiency [44,45]. This modulation enables the bacterium to swiftly adjust to variations in host iron levels, enhancing the expression of virulence determinants (biofilm, extracellular enzymes, EPSs) and resilience to oxidative stress. Dysregulation of iron homeostasis or inhibition of siderophore systems leads to attenuation of virulence and reduced colonization and infection capacity [39,42].

The type VI secretion system (T6SS) is essential in the pathogenicity and competitive interactions of the *Citrobacter freundii* complex, exhibiting an extensive genomic distribution and diversity in toxic effectors that enhance its virulence and adaptability in different environments [46]. Hemolysins and phospholipases, identified as T6SS effectors, contribute to cell lysis and nutrient acquisition, underscoring their roles in increasing the severity of infection and treatment challenges [47,48,49].

The *Citrobacter freundii* complex uses immune evasion tactics primarily through lipopolysaccharide (LPS) variability and mechanisms that confer resistance to complement and phagocytosis [50]. By altering the structural constituents of lipopolysaccharides (LPS), specifically the O antigen and lipid A moieties, the bacterium reduces the activation of the toll-like receptor 4 (TLR4), consequently diminishing the inflammatory responses of the host [51]. Furthermore, the existence of a polysaccharide capsule along with its interactions with complement regulatory proteins further obstruct opsonization, thereby enhancing the bacterium’s survival within the host immune milieu [51,52,53,54].

Biofilm-associated pathophysiology in infections caused by the *Citrobacter freundii* complex encompasses surface-attached microbial consortia that are shielded by an extracellular polymeric substance (EPS), which augments bacterial resistance to antibiotics and immune system responses, consequently resulting in persistent infections and associated complications [55,56]. Interactions among multiple microbial species, particularly with Enterococcus and Proteus spp., intensify the severity of the disease by amplifying biofilm biomass and antibiotic resistance, thereby facilitating catheter-related urinary tract infections and fostering colonization by *Citrobacter* spp. [57,58].

Table 1 outlines virulence factors in the *Citrobacter freundii* complex, detailing confirmed and predicted functions alongside genomic context. These factors facilitate host invasion, immune evasion, biofilm formation, iron acquisition, cytotoxicity, and multidrug resistance, highlighting the clinical significance and adaptability of this pathogen.

## 5. Antimicrobial Resistance

The antimicrobial resistance mechanisms of *Citrobacter freundii* complex elucidate treatment challenges and guide antibiotic selection. These mechanisms are categorized into 4 types:

a. Intrinsic resistance. Basal production of AmpC in *C. freundii* confers intrinsic resistance to aminopenicillins, amoxicillin–clavulanate, ampicillin–sulbactam, and first- and second-generation cephalosporins, rendering them ineffective [59,60].

b. Inducible AmpC expression. *C. freundii* exhibits a moderate to high risk for AmpC derepression during third-generation cephalosporin therapy, with exposure potentially increasing MICs and leading to treatment failure. Resistance can arise rapidly, complicating clinical management, prompting avoidance of third-generation cephalosporins in most cases [60,61].

c. Additional β-Lactam resistance mechanisms. *C. freundii* utilizes various mechanisms for β-lactam resistance, including alterations in outer membrane proteins and porin loss, which reduce drug permeability and enhance resistance through combined effects with AmpC. Efflux pumps are overexpressed, actively expelling β-lactams and modulating antibiotic efficacy, especially affecting specific agents. *C. freundii* can acquire various β-lactamases, including ESBLs and carbapenemases, further complicating resistance profiles and posing a significant threat. Overexpression of efflux pumps (e.g., AcrAB-like systems) contributes to resistance by actively expelling β-lactams from the periplasmic space [60].

d. Resistance to non-β-lactam agents. *C. freundii* exhibits variable resistance to non-β-lactam antibiotics: fluoroquinolones (resistance develops primarily through mutations in DNA gyrase and topoisomerase IV genes), with mutations at codons 83 and 87 of gyrA and codon 80 of parC conferring high-level resistance. Efflux pump overexpression can modulate final MICs, aminoglycosides (resistance is mediated by aminoglycoside-modifying enzymes encoded on integrons and plasmids), trimethoprim–sulfamethoxazole (resistance is common, often mediated by integron-encoded genes), and carbapenems, fourth-generation cephalosporins (cefepime), amikacin, and fluoroquinolones that remain reliable agents for multidrug-resistant *C. freundii* when susceptibility is confirmed [59,60,61].

The molecular regulation of intrinsic β-lactam resistance in the *Citrobacter freundii* complex relies on the inducible expression of the AmpC β-lactamase, controlled by the ampR-ampC-ampD-ampG network. In the basal state, AmpC is expressed at low levels, but exposure to β-lactams or disruption of peptidoglycan recycling leads to rapid induction [59,60,61].

AmpR encodes a LysR-type regulator that, in the absence of stimulus, binds UDP-MurNAc-pentapeptide and inhibits AmpC transcription. Accumulation of cytosolic muropeptides (1,6-anhydroMurNAc-peptides) following the action of β-lactams or derepressive mutations causes AmpR to activate AmpC transcription [61,62,63,64]. AmpG encodes a permease essential for the transport of muropeptides from the periplasm to the cytosol, facilitating signaling to AmpR [60,61]. AmpD encodes a cytosolic amidase that degrades muropeptides; inactivating mutations in AmpD lead to persistent accumulation of muropeptides and stable derepression of AmpC, with constitutive hyperproduction [65,66,67].

Derepressive mechanisms include point mutations or deletions in AmpD, AmpR, and AmpG, as well as promoter or attenuator variations in AmpC, which increase transcription independent of β-lactam stimulus [65,68]. The G102E mutation in AmpR, for example, causes constitutive activation of AmpC, regardless of the presence of muropeptides or AmpG function [63,69,70]. These molecular changes explain the rapid emergence of resistance to third-generation cephalosporins during treatment, which is why the Infectious Diseases Society of America recommends avoiding monotherapy with these antibiotics in Enterobacterales infections with inducible AmpC [68,71].

The main acquired resistance genes identified in the *Citrobacter freundii* complex include:

1. Plasmid-mediated extended-spectrum β-lactamases: The blaCTX-M (especially CTX-M-15 and CTX-M-9), blaSHV (e.g., SHV-12), and blaTEM genes are frequently detected, often located on class 1 integrons or conjugative plasmids, facilitating inter- and intra-species dissemination [72,73].
2. Carbapenemases: The *Citrobacter freundii* complex can acquire the genes blaNDM-1 (New Delhi metallo-β-lactamase), blaKPC-2 (*Klebsiella pneumoniae* carbapenemase), blaOXA-48, and the OXA-181/OXA-1186 variants (OXA-48-type oxacillinases), as well as blaVIM-1 and blaVIM-2 (Verona integron-encoded metallo-β-lactamase). These genes are located on transferable plasmids (IncX3, IncP6, IncN, etc.) and can coexist in the same isolate, generating extended resistance to carbapenems and other β-lactams [73,74,75].
3. Plasmid-mediated quinolone resistance genes (PMQR): The qnrB family is particularly common in Citrobacter, with multiple alleles (qnrB1, qnrB2, qnrB4, qnrB62, etc.) identified on both chromosomes and plasmids. Citrobacter spp. are the main source of qnrB in Enterobacterales, and plasmid transfer is documented. Other PMQR genes include qnrS1, aac(6′)-Ib-cr, and oqxAB, but qnrB predominates [76,77,78,79,80].

Horizontal gene transfer (HGT) networks play a central role in the spread of antibiotic resistance within the *Citrobacter freundii* complex, facilitating the rapid mobilization of resistance genes between species and lineages of Enterobacteriaceae, including *Enterobacter* and *Klebsiella*. Common backbone conjugative plasmids, such as IncC, IncM2, IncP6, IncHI1A, and IncN, are frequently identified in *Citrobacter* isolated from wastewater and hospital environments, with the ability to transfer critical resistance genes (e.g., blaKPC, blaNDM, blaOXA-48, mcr-9, qnrB) between *Citrobacter*, *Enterobacter* and *Klebsiella*, but also to other families of Gammaproteobacteria [24,81,82,83,84].

These plasmids display conserved structures, with mobile elements (class 1 integrons, IS26 transposons, ISEcp1) that facilitate the integration and transfer of genes conferring extended resistance to β-lactams, carbapenems, and quinolones [85,86]. Genomic studies demonstrate the existence of dense networks of plasmid-host interactions in wastewater communities, where plasmids with resistance genes have a wide host range and are essential for the connectivity of the microbial network [84].

Wastewater systems and wastewater treatment plants enhance horizontal gene transfer by creating environments with high bacterial densities, the presence of antibiotics, biocides, and heavy metals at sub-inhibitory concentrations, which stimulate conjugation, transformation, and transduction [87,88,89]. Conjugation dominates as a mechanism of HGT, with increased frequencies in biofilms and anaerobic environments, and biosolids and post-disinfection effluents can contain transferable multidrug-resistant plasmids without detectable fitness cost [90,91,92]. Thus, wastewaters act as hotspots for HGT, favoring the emergence and rapid dissemination of multidrug-resistant strains of the *Citrobacter freundii* complex, with plasmids shared with *Enterobacter* and *Klebsiella* [82,83,90]. These processes require epidemiological surveillance and advanced wastewater management to limit the spread of antibiotic resistance.

In the *Citrobacter freundii* complex, the fitness cost associated with antibiotic resistance, including β-lactam resistance, is determined both by the nature of the genetic determinant (chromosomal mutations vs. plasmid-acquired genes) and by the bacterial capacity for metabolic compensation. From a systems biology perspective, resistance acquired through transferable genes (e.g., AmpC β-lactamases, ESBLs, carbapenemases) imposes a lower fitness cost compared to mutations in essential genes, because the integration of plasmids with resistance genes disrupts central metabolic networks less [93,94].

Metabolic compensation is achieved by reconfiguring metabolic fluxes: the bacterial transcriptome exposed to β-lactams exhibits upregulation of genes involved in amino acid catabolism, fermentation, membrane biosynthesis, and oxidative stress response, as well as activation of transport and efflux systems [95,96]. These adaptations allow for the maintenance of growth and survival in the presence of antibiotics, reducing the energetic cost of resistance. For example, overproduction of AmpC or ESBL β-lactamases is associated with increased expression of peptidoglycan recycling genes and efflux systems, with minimal impact on fitness in the absence of antibiotics [97,98].

However, derepressive mutations or those affecting membrane permeability can impose significant metabolic costs, manifested by decreased growth rate and competitiveness, especially in environments without antibiotic pressure. These costs can be compensated by secondary mutations or metabolic reconfigurations, including increased respiratory and fermentative flux [99,100].

Recent studies indicate a global increase in acquired resistance genes within the *Citrobacter freundii* complex, showing diverse geographic patterns and significant epidemiological concerns in both hospital and community settings. The prevalence of plasmid-mediated ESBL genes and carbapenemases, together with the co-occurrence of PMQR genes, underscores the urgent need for genomic and molecular surveillance to mitigate the spread of multidrug-resistant strains [4,78,101,102,103,104].

## 6. Clinical Manifestations and Management

It is important to note that infections caused by *Citrobacter freundii* complex (CFC) do not exhibit unique clinical manifestations that distinguish them from other *Enterobacterales*, such as *Enterobacter* species. These infections are predominantly hospital-acquired (nosocomial) and occur most frequently in patients with significant comorbidities or those exposed to invasive medical devices. Clinical outcomes for CFC infections are generally favorable provided that appropriate antimicrobial therapy is administered promptly [5,105].

Clinical manifestations associated with *Citrobacter freundii* complex include predominantly urinary tract infections (cystitis, pyelonephritis, asymptomatic bacteriuria), complicated urinary tract infections (associated with urogenital anomalies, catheters, comorbidities, or nosocomial infections), and biliary tract infections (cholangitis, cholecystitis), often in a polymicrobial context or in patients with abdominal procedures or risk factors [3,5,105,106].

Urinary tract infections with *Citrobacter freundii* complex frequently occur in the elderly, children with urogenital anomalies, hospitalized patients, or those with exposure to medical devices. Emerging phenotypes include multidrug-resistant (MDR) strains, producing ESBLs and carbapenemases (NDM, KPC, OXA-48), which cause extensive resistance to third-generation cephalosporins and carbapenems, complicating empirical treatment and increasing the risk of therapeutic failure [9,107,108,109].

Complicated urinary tract infections are manifested by fever, dysuria, back pain, hematuria, pyuria, and may progress to pyelonephritis, urosepsis, or bacteremia, especially in vulnerable hosts (immunosuppressed, diabetic, patients with urinary catheters) [110,111,112]. Biliary tract infections with *Citrobacter freundii* complex typically occur after surgery or in patients with gallstones, are often polymicrobial, and are associated with severe disease [113].

Emerging phenotypes include strains with increased biofilm formation capacity, persistence in hospital environments (sinks, drains), and adaptation to antimicrobial selective pressures, which favors nosocomial transmission and infection outbreaks [9,10,109,114].

Clinical manifestations of *Citrobacter freundii* complex in neonatal intensive care unit outbreaks include neonatal sepsis, meningitis, central nervous system (CNS) infections, bacteremia, pneumonia, urinary tract infections, conjunctivitis, and surgical wound infections. Most commonly, the onset is bacteremia, followed by respiratory and gastrointestinal involvement; meningitis may progress to brain abscess, although the incidence of abscess is lower than with *Citrobacter koseri* [115,116,117,118]. Emerging phenotypes include multidrug-resistant (MDR) strains with resistance to cephalosporins, carbapenems, and quinolones, including ESBLs and carbapenemases, which complicates empirical treatment and increases mortality [1,109,116,119]. Outbreak strains exhibit increased adherence, biofilm formation, and can activate the NLRP3 inflammasome via the type VI secretion system (T6SS), promoting cytotoxicity and immune evasion [120]. Risk factors for bacteremia include prematurity, low birth weight, immune immaturity, exposure to invasive devices (catheters, ventilation), comorbidities, prolonged hospitalization, gastrointestinal or respiratory colonization, and contact with healthcare personnel or contaminated environments (sinks, drains) [1,115,119].

In immunocompromised hosts, the *Citrobacter freundii* complex exhibits unique pathogenic behavior: intracellular invasion and replication in brain endothelial cells, biofilm formation, inflammasome activation, and persistence in hospital settings, with predominantly indirect transmission from environmental reservoirs [9,109,118]. The prognosis is worsened by MDR and the difficulty of epidemiological control.

Treatment requires antibiotic therapy tailored to local susceptibility, with preference for carbapenems, aminoglycosides, or tigecycline, according to susceptibility, and strict infection control measures [1,116,119].

The Infectious Diseases Society of America recommends cefepime as a preferred agent for Citrobacter freundii complex because cefepime is a weak inducer of AmpC beta-lactamase and is structurally resistant to AmpC hydrolysis, due to its ability to form stable acyl–enzyme complexes. This minimizes the risk of resistance emergence during therapy and allows cefepime to retain activity against AmpC-producing organisms, including *C. freundii*, when the isolate is susceptible [68,71].

Newer beta-lactam/beta-lactamase inhibitor combinations such as ceftazidime–avibactam should be reserved for multidrug-resistant Citrobacter freundii complex infections, particularly those exhibiting carbapenem resistance. The Infectious Diseases Society of America advises against routine use of these agents for standard AmpC-E infections, as their activity is best preserved for cases where carbapenem resistance is present or other options are limited. While ceftazidime–avibactam is effective against AmpC-E, resistance can emerge, and its use should be prioritized for organisms with limited susceptibility profiles [68,71].

## 7. Diagnostic Gaps

Current diagnostic gaps in distinguishing *Citrobacter freundii* complex from Enterobacter species are primarily due to phenotypic overlap and limitations of MALDI-TOF MS. Both genera share similar colony morphology, biochemical profiles, and inducible AmpC β-lactamase activity, leading to frequent misidentification in routine clinical workflows [114,121]. MALDI-TOF MS, while rapid and widely adopted, is constrained by incomplete reference databases and insufficient discriminatory spectral markers for less common or newly described *Citrobacter* species, resulting in species-level misidentification rates of up to 16–45% for *Citrobacter* and *Enterobacter*, especially in complex or atypical isolates [122,123,124]. Even with expanded spectral libraries and machine learning algorithms, reliable separation of *Citrobacter freundii* complex from Enterobacter cloacae complex remains challenging, particularly for environmental or multidrug-resistant strains [125].

Whole-genome sequencing (WGS) is currently the only tool with sufficient resolution to definitively distinguish *Citrobacter freundii* complex from Enterobacter species. WGS enables high-resolution taxonomic assignment using average nucleotide identity (ANI), core genome MLST, and phylogenomic analysis, overcoming the limitations of phenotypic and proteomic methods [104,109,126,127]. Recently, cgMLST and wgMLST schemes have been validated for *Citrobacter freundii* and related species, providing robust discrimination and supporting outbreak investigations and surveillance [109,126].

Development of molecular tests targeting unique markers of *Citrobacter freundii* complex is ongoing, but not yet standardized for clinical use. Some PCR assays and molecular panels have targeted species-specific β-lactamase genes (e.g., chromosomal AmpC variants, novel CMY alleles) and virulence loci, but these are not universally adopted and may lack sensitivity for all complex members [104,128]. The open pan-genome and frequent horizontal gene transfer further complicate marker selection [104,128]. No commercial rapid molecular test currently offers reliable, routine discrimination of *Citrobacter freundii* complex from Enterobacter species based on unique genetic markers.

## 8. Future Directions

Key future directions and knowledge gaps regarding the *Citrobacter freundii* complex include:

Factors driving species divergence: The processes underlying divergence within this complex are significantly influenced by extensive occurrences of gene acquisition and loss, horizontal gene transfer (HGT), as well as a pangenome architecture characterized by a considerable proportion of uncharacterized accessory genes. Analyses of average nucleotide identity (ANI) in conjunction with phylogenomic investigations have unveiled the presence of no fewer than six distinct species and various genomic alterations; however, the taxonomic framework remains deficient, plagued by recurrent classification inaccuracies and an absence of functional criteria requisite for species delineation [18,109].

The need for structure–function studies for AmpC and other beta-lactamases: Despite the identification of derepressive mutations and novel variants of AmpC (such as CMY-41 and CMY-N106S), the comprehensive understanding of enzymatic functionality, substrate specificity, and the implications for clinical resistance remains insufficiently characterized. Structure–function analyses are imperative for elucidating the evolution and efficacy of β-lactam hydrolysis, in addition to facilitating the development of targeted inhibitors [128,129,130].

Identification of novel virulence determinants: Comparative genomics has revealed virulence islands, type VI secretion systems, adhesins, toxins, and siderophore clusters; however, the functions of many accessory genes remain unknown. Functional and phenotypic studies are needed to correlate molecular determinants with clinical pathogenicity and tissue tropism [36,104].

The role of long-read sequencing in resolving plasmid structures: Long-read sequencing (Nanopore, PacBio) allows for the complete assembly of genomes and plasmids, the identification of complex structures, mobile elements, and resistance genes, overcoming the limitations of short-read sequencing. This is crucial for monitoring the transmission of MDR plasmids and for epidemiological surveillance [131,132].

Significance of environmental monitoring: Environmental reservoirs (such as wastewater, sinks, and soil) serve as significant sources of multidrug-resistant *Citrobacter* and associated resistance genes, posing a risk of transmission to humans. Current genomic monitoring of these reservoirs is inadequate, although recent investigations reveal a substantial diversity of multidrug-resistant species and lineages within aquatic and wildlife habitats, underscoring the necessity of incorporating a One Health approach into monitoring efforts [24,81,104]. These factors are of paramount importance for research, monitoring, and epidemiological management of the *Citrobacter freundii* complex.

## 9. Conclusions

The *Citrobacter freundii* complex (CFC) is increasingly acknowledged as an emerging nosocomial pathogen with considerable clinical ramifications, attributable to its genomic adaptability, a wide array of virulence factors, and swiftly advancing mechanisms of antimicrobial resistance. CFC is progressively associated with urinary tract infections, bloodstream infections, and outbreaks within healthcare facilities, with environmental reservoirs such as sinks and wastewater serving a pivotal role in the facilitation of transmission. Prospective research endeavors should concentrate on delineating species boundaries, conducting functional analyses of resistance and virulence determinants, employing long-read sequencing to elucidate plasmid architecture, and implementing extensive environmental surveillance to address overlooked reservoirs and enhance infection prevention methodologies.

## Figures and Tables

**Table 1:** The table lists major virulence factors identified in the *Citrobacter freundii* complex. These factors contribute to host invasion, immune evasion, biofilm formation, iron acquisition, cytotoxicity, and multidrug resistance, underpinning the clinical relevance and adaptability of this emerging pathogen.

Virulence Factor	Known/Predicted Function	Genomic Location/Cluster
Type VI Secretion System (T6SS)	Interbacterial competition, cytotoxicity	Genomic island
Fimbriae (e.g., FimH, Type 1)	Adhesion to host cells, biofilm formation	Genomic islands
Pili (Type IV)	Surface attachment, biofilm formation	Genomic islands
Enterobactin biosynthesis	Iron acquisition, survival in the host	Chromosomal cluster
Aerobactin biosynthesis	Iron acquisition (predicted)	Genomic cluster
Biofilm formation	EPS production, persistence	Chromosomal
Capsule synthesis	Immune evasion, complement resistance	Genomic islands
LPS modification (PmrA/PmrB)	Colistin resistance, immune evasion	Chromosomal

## Data Availability

No new data were created or analyzed in this study. Data sharing is not applicable to this article.

## Abbreviations

The following abbreviations are used in this manuscript:

ESBLs	extended-spectrum beta-lactamases
*E. coli*	*Escherichia coli*
MALDI-TOF MS	Matrix-Assisted Laser Desorption Ionization Time-of-Flight Mass Spectrometry
WGS	whole-genome sequencing
ANI	average nucleotide identity
dDDH	digital DNA-DNA hybridization
MLSA	multilocus sequence analysis
EPSs	extracellular polymeric substances
LPS	lipopolysaccharide
TLR4	toll-like receptor 4
T6SS	type VI secretion system
Fur	ferric uptake regulator
PMQR	plasmid-mediated quinolone resistance genes
HGT	horizontal gene transfer
MDR	multidrug-resistant
CNS	central nervous system
CFC	Citrobacter freundii complex
UTI	Urinary tract infection
dDDH	Digital deoxyribonucleic acid–deoxyribonucleic acid hybridization
16S rRNA	16S ribosomal ribonucleic acid gene
eDNA	Extracellular deoxyribonucleic acid
O antigen	O-polysaccharide antigen
qnrB	Quinolone resistance gene family
IS26	Insertion sequence 26
ISEcp1	Insertion sequence associated with blaCTX-M mobilization
ICU	Intensive care unit
NLRP3	NLR family pyrin domain containing 3
cgMLST	Core genome multilocus sequence typing
wgMLST	Whole-genome multilocus sequence typing
blaNDM-1	Gene encoding New Delhi metallo-beta-lactamase 1
